# Rituximab-Associated Acute Ischemic Stroke in a Patient With Primary Angiitis of the Central Nervous System

**DOI:** 10.7759/cureus.71412

**Published:** 2024-10-14

**Authors:** Furkan Asan, Zeynep Esin Sayar, Kagan Gokdeniz Karadeniz, Bengi Gul Turk, Sabahattin Saip

**Affiliations:** 1 Department of Neurology, Istanbul University-Cerrahpasa, Cerrahpasa School of Medicine, Istanbul, TUR

**Keywords:** acute ischemia, central nervous system vasculitis, cerebrovascular disease, rituximab, rituximab-induced stroke, vasospasm

## Abstract

During or after rituximab treatment, various serious side effects may occur. Cerebrovascular diseases are relatively limited among these side effects, and whether they are contingent on rituximab treatment is unclear. This case report highlights an unusual and severe event after rituximab treatment. Our patient was a 32-year-old female diagnosed with primary angiitis of the central nervous system (PACNS). Despite meticulous management with azathioprine, the patient's condition exhibited persistent ischemic activity, necessitating a therapeutic shift to rituximab. Regrettably, very soon after the initial rituximab infusion, a substantial acute ischemic stroke took place in the right cerebral hemisphere. We have interpreted that the significant stenosis in the right internal carotid artery, which had developed due to PACNS, may pose a risk for the development of acute ischemia in our patient after rituximab treatment. In conclusion, careful evaluation of rituximab treatment may be appropriate in patients with serious stenosis of intracranial major arteries.

## Introduction

Primary angiitis of the central nervous system (PACNS) poses significant challenges in management, often requiring immunosuppressive agents such as rituximab, azathioprine, and cyclophosphamide. Rituximab, an anti-CD20 monoclonal antibody, is used to treat various lymphoproliferative, rheumatologic, and neurological diseases. The most common side effects during or immediately after rituximab infusion are infusion-related reactions (IRR). IRR occurs within the first 24 hours after infusion and may present with symptoms such as fever, rash, itching, nausea, headache, hypotension, bronchospasm, and angioedema. The frequency of the IRR varies according to indication, but the probability is highest in the first infusion and decreases with subsequent infusions [1].

There are reported cases of myocardial infarction (MI) and vasospasm associated with IRR [2]. However, according to our literature review, there are no documented cases of ischemic stroke occurring within the first 24 hours following infusion. Therefore, we wanted to report our case, which we followed due to an ischemic stroke that occurred within a few hours after the completion of the infusion.

## Case presentation

The 32-year-old female had a four-year history of diagnosis with PACNS. Five years ago, the patient was admitted to our clinic because of recurrent transient ischemic attacks (TIAs) with right-sided hemihypoesthesia. During these episodes, the patient reported experiencing reduced sensation in the entire right half of his body, including her face. These episodes have been occurring for about a year, happening four to five times per month, with durations varying from 10 minutes to two hours.

**Figure 1:**
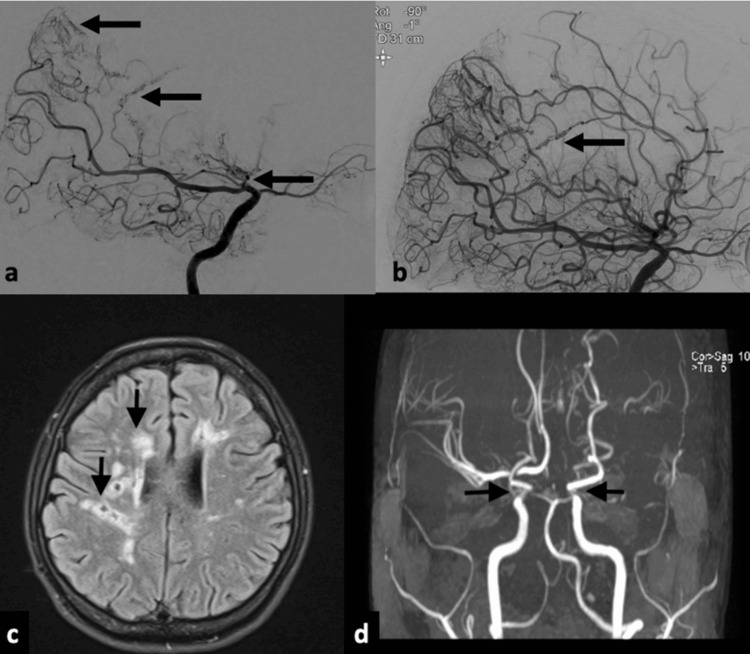
(a,b) Digital subtraction angiography of the patient demonstrates occlusion of the right ICA and collateral flow from posterior cerebral artery branches to the middle and anterior cerebral arteries (black arrows). (c) Brain MRI before the rituximab treatment showed multiple, small, chronic, ischemic foci of white matter (black arrows). (d) Brain TOF MRI angiography revealed severe stenosis of the right ICA and total occlusion of left ICA at the supraclinoid segment (black arrows) MRI: magnetic resonance imaging; TOF: time-of-flight; ICA: internal carotid artery

Neuroimaging studies revealed bilateral, multiple, and subcortical white matter ischemic foci and severe stenosis in the supraclinoid segment of the left internal carotid artery (ICA). Digital subtraction angiography (DSA) demonstrated an occlusion at the supraclinoid level of the left ICA, with the pial-pial collateral flow from the posterior cerebral artery to the left middle and anterior cerebral artery (Figures 1a, 1b). Additionally, concentric contrast enhancement of the supraclinoid segment of the left ICA was noted on vessel wall imaging with magnetic resonance imaging (MRI). No pathological findings were detected in the rheumatological marker levels, including antinuclear antibody, antineutrophil cytoplasmic antibodies, perinuclear antineutrophil cytoplasmic antibodies, anti-double-stranded DNA, C3, C4, rheumatoid factor, and anticyclic citrullinated peptides. Cerebrospinal fluid examination revealed normal protein levels without any cell reaction.

Despite the terminal ICA involvement being unilateral, moyamoya disease and PACNS were among our possible preliminary diagnoses. However, the presence of pial-pial anastomoses originating from the posterior cerebral artery rather than anastomoses from external carotid branches or anastomoses at the base of the brain made moyamoya diseases less likely. With the contrast enhancement on the vessel wall MRI, the patient was diagnosed with PACNS. Treatment was initiated with oral methylprednisolone and azathioprine. Oral methylprednisolone was tapered off within three months, and treatment was continued with azathioprine alone.

Two years later, treatment was discontinued due to pregnancy. After two years without hospital admission, the patient was readmitted to our clinic because of recurrent TIAs characterized by left hemiplegia and hemihypoesthesia. MRI revealed a marked increase in chronic ischemic lesions in the right hemisphere and severe stenosis of the right ICA at the supraclinoid segment (Figures 1c, 1d). Due to severely progressed disease activity, it was decided to start the treatment with rituximab. Before rituximab infusion, premedication with intravenous pheniramine (45.5 mg), methylprednisolone (100 mg), and paracetamol (500 mg) had been administered. Subsequently, a total dose of 1,000 mg of rituximab had been infused over six hours. During the infusion, the patient developed disseminated rash and itching at the neck and throat. These findings were considered as IRRs. Rituximab infusion was interrupted, and an additional dose of pheniramine was administered, leading to the disappearance of symptoms within an hour. The patient's vital signs, including blood pressure, remained normal throughout the infusion.

**Figure 2:**
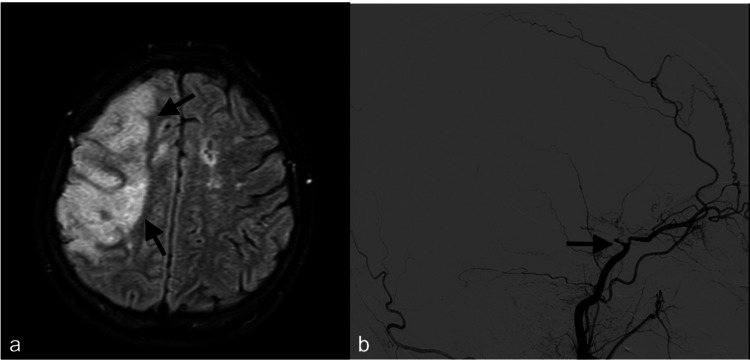
(a) Extensive acute ischemia at the right hemisphere (black arrows). (b) Digital subtraction angiography of the patient had revealed the total occlusion of the right ICA after giving off the ophthalmic artery (black arrow) ICA: internal carotid artery

**Figure 3:**
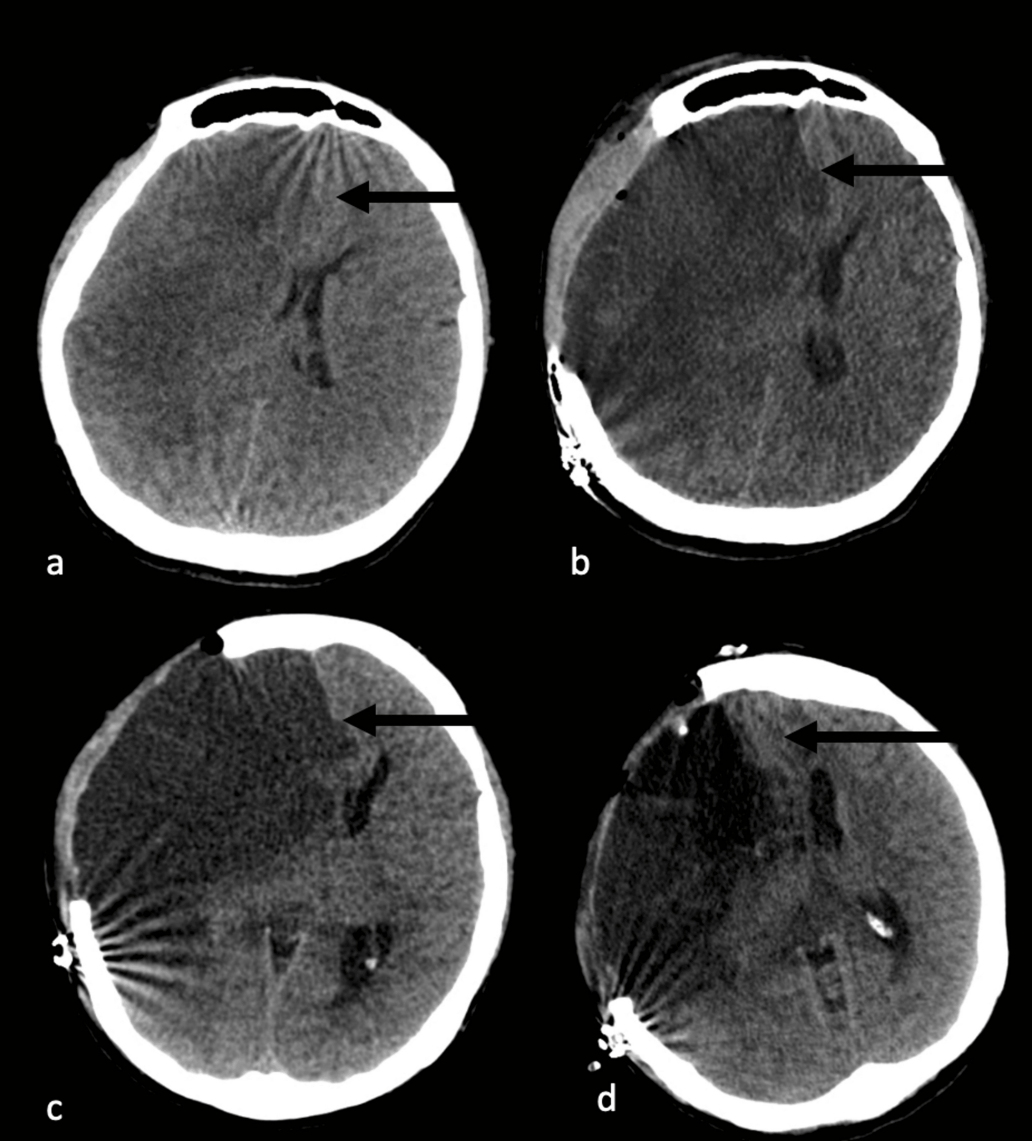
Serial brain computed tomographies of the patient (a) before decompressive craniotomy, (b) 48 hours after craniotomy, (c) 72 hours after craniotomy, and (d) 48 hours after partial frontal lobectomy (black arrows)

After four hours of the end of the rituximab infusion, the patient developed acute left hemiplegia and hemihypoesthesia. Initially, she attributed these symptoms to recurrent TIAs she had previously experienced on several occasions. However, as the weakness persisted for several hours, further evaluation, which was performed after the patient's call to the medical team, revealed an extensive acute ischemic lesion in the right cerebral hemisphere (Figure 2a). DSA demonstrated total occlusion of the right ICA after giving off the ophthalmic artery, with no contrast filling in the right middle and anterior cerebral arteries (Figure 2b). The left middle and anterior cerebral arteries still had blood flow via collateral branches from the posterior cerebral artery. Over the following days, malignant cerebral edema developed, and decompressive surgery was performed (Figures 3a, 3b). Despite the surgery, the patient's consciousness continued to deteriorate. Partial frontal lobectomy was performed because of the low Glasgow Coma Scale score (12 out of 15) and persistence of midline shift in the control computed tomography scan 72 hours later (Figure 3c). After the second operation, the midline shift and herniation resolved (Figure 3d). After one year of the event, the patient was still left hemiplegic.

## Discussion

A comprehensive review of the literature at PubMed reveals a scarcity of reported cases involving ischemic strokes after rituximab infusions. On the other hand, the rituximab product monograph reports that ischemic stroke occurred in four patients with hematologic malignancies after infusion of the drug but at an unspecified interval [3]. However, those patients were treated simultaneously with cyclophosphamide, doxorubicin, methylprednisolone, and vincristine. It is also reported that all of those patients were above 70 years old and had concurrent various risk factors for cardiovascular diseases. Additionally, one patient with rheumatoid arthritis who received rituximab developed ischemic stroke five months after infusion [4]. No cases of ischemic stroke within 24 hours of infusion have been previously reported.

IRR encompasses hypersensitivity reactions and cytokine-releasing syndrome (CRS). In CRS, releasing cytokines from various sources can lead to symptoms such as headache, nausea, tachycardia, hypotension, and shortness of breath. Although symptoms are typically self-limiting, they can occasionally be life-threatening. The simultaneous release of different cytokines may result in various cardiovascular toxicities, including hypotension, MI, and arrhythmias [5]. Furthermore, coronary vasospasm without intra-arterial thrombosis had been reported during rituximab infusion exceptionally [6,7]. Similarly, the ischemic stroke in our patient may have been caused by vasospasm following rituximab infusion.

Our case raises a question about the relationship between the severe ischemic event and the rituximab infusion. The ischemic stroke of our patient developed in the territory of the ICA with critical stenosis, which was symptomatic even before the infusion. Therefore, the causality of rituximab can be questioned. However, the absence of any signs of such severe and widespread ischemia during the long years of disease history of the patient and the development of ischemia in such close temporal proximity have led to considering rituximab infusion as a potential trigger. Furthermore, the ischemic stroke occurred in the right hemisphere, which was outstandingly poor in terms of collaterals. No symptomatic or asymptomatic acute ischemia has been detected in the territories of the left middle and anterior cerebral arteries, which were supplied through well-developed collaterals.

It has been reported that there is an increase in serum interleukin (IL)-6, IL-8, and tumor necrosis factor-alpha levels in rituximab-related CRS [8]. These increases were more pronounced in patients with clinically detected IRR. It is known from studies conducted in patients with subarachnoid hemorrhage that IL-6 has an important role in evolving the vasospasm in cerebral vasculature [9]. Considering these data, in our patient who had a low-grade IRR during the infusion, even the modest level of vasospasm that may have developed in the severely stenotic ICA may have caused total occlusion of the artery and extensive ischemia. However, a definitive conclusion cannot be drawn.

This case presents a unique scenario where rituximab infusion somehow probably triggered a severe ischemic event. While previous reports of few ischemic stroke events after rituximab infusion are predominantly with hematological malignancies, our case prompts a reconsideration of rituximab use in individuals with existing intra/extracranial arterial stenosis or compromised cerebral perfusion.

## Conclusions

Rituximab is a monoclonal antibody that is used frequently in different medical disciplines for various indications. This report underscores the critical importance of vigilance when considering rituximab therapy, particularly in patients with documented severe stenosis of intracranial arteries. While our case may not conclusively establish a direct causal relationship between rituximab and the ischemic event, it highlights the need for cautious evaluation in patients with severely compromised cerebral vasculature.

## References

[1] Paul F, Cartron G (2019). Infusion-related reactions to rituximab: frequency, mechanisms and predictors. Expert Rev Clin Immunol.

[2] Gogia A, Khurana S, Paramanik R (2014). Acute myocardial infarction after first dose of rituximab infusion. Turk J Haematol.

[3] (2024). Rituximab package insert (Biogen Idec Inc., and Genentech, Inc.). http://www.accessdata.fda.gov/drugsatfda_docs/label/2011/103705s5344lbl.pdf.

[4] Emery P, Fleischmann R, Filipowicz-Sosnowska A (2006). The efficacy and safety of rituximab in patients with active rheumatoid arthritis despite methotrexate treatment: results of a phase IIB randomized, double-blind, placebo-controlled, dose-ranging trial. Arthritis Rheum.

[5] Baik AH, Oluwole OO, Johnson DB, Shah N, Salem JE, Tsai KK, Moslehi JJ (2021). Mechanisms of cardiovascular toxicities associated with immunotherapies. Circ Res.

[6] Lee L, Kukreti V (2012). Rituximab-induced coronary vasospasm. Case Rep Hematol.

[7] Ke C, Khosla A, Davis MK, Hague C, Toma M (2015). A case of coronary vasospasm after repeat rituximab infusion. Case Rep Cardiol.

[8] Winkler U, Jensen M, Manzke O, Schulz H, Diehl V, Engert A (1999). Cytokine-release syndrome in patients with B-cell chronic lymphocytic leukemia and high lymphocyte counts after treatment with an anti-CD20 monoclonal antibody (rituximab, IDEC-C2B8). Blood.

[9] Lucke-Wold B, Hosaka K, Dodd W, Motwani K, Laurent D, Martinez M, Hoh B (2021). Interleukin-6: important mediator of vasospasm following subarachnoid hemorrhage. Curr Neurovasc Res.

